# Genomic analysis and therapeutic efficacy evaluation of bacteriophage PK2420 for pneumonia caused by hypervirulent *Klebsiella pneumoniae* (K20 serotype)

**DOI:** 10.1128/msystems.01632-24

**Published:** 2025-04-16

**Authors:** Jinfeng Chen, Junxia Feng, Xiaohu Cui, Lijuan Huang, Bing Du, Yuyan Xia, Guanhua Xue, Yanling Feng, Yuehua Ke, Hanqing Zhao, Jinghua Cui, Chao Yan, Lin Gan, Zheng Fan, Tongtong Fu, Ziying Xu, Yang Yang, Zihui Yu, Shuo Zhao, Zhen Wang, Yiming Kong, Boyi Jiang, Mingxuan Wang, Mengyao Ling, Jing Yuan

**Affiliations:** 1 Capital Institute of Pediatrics-Peking University Teaching Hospital, Beijing, China; 2 Department of Bacteriology, Capital Institute of Pediatrics 36776, Beijing, China; 3 Children’s Hospital Capital Institute of Pediatrics, Chinese Academy of Medical Sciences & Peking Union Medical College, Beijing, China; University of Michigan, Ann Arbor, Michigan, USA

**Keywords:** hypervirulent *Klebsiella pneumoniae*, K20 serotype, bacteriophage therapy

## Abstract

Hypervirulent *Klebsiella pneumoniae* (hvKp) strains are increasingly recognized for their aggressive nature, which leads to severe clinical outcomes. The emergence of multidrug-resistant strains constitutes a substantial challenge for clinical management. Phage therapy offers a potential solution to the antibiotic resistance crisis. A multidrug-resistant hvKp strain, K2420 (K20 serotype), was used to isolate bacteriophages from hospital sewage. Phage morphology, biological properties, and genome characteristics were analyzed using transmission electron microscopy, plaque assays, and whole-genome sequencing. Therapeutic safety and efficacy were assessed in an acute pneumonia murine model induced by intratracheal injection of K2420. Assessment parameters included bacterial load, phage titer, body temperature, cytokine levels, histopathological findings, and other relevant indicators. Phage PK2420, a member of the *Autographiviridae* family and *Przondovirus* genus, was identified. It rapidly lyses *K. pneumoniae* (K20 serotype), inhibits biofilm formation, and exhibits a burst size of 37.4 plaque-forming units/cell. The phage is stable at temperatures ranging from 0°C to 40°C and pH values between 6 and 9. Its genome, 41,155 bp in length, contains 46 coding sequences. The phage has no genes associated with antibiotic resistance, virulence, or lysogeny. *In vivo*, PK2420 substantially reduced *K. pneumoniae* bacterial loads, improved survival rates, and alleviated pneumonia severity without observable side effects. Phage PK2420 exhibits lytic activity against *K. pneumoniae* both *in vitro* and in murine models, providing a promising and safe option for the treatment of hvKp infections.

**IMPORTANCE:**

Our investigation provides insights into the interaction mechanism among hypervirulent *Klebsiella pneumoniae* (hvKp) (K20 serotype), phage, and the host in a mouse pneumonia model, offering a valuable reference for future research on phage pharmacokinetics. This study demonstrated that bacteriophage PK2420 exhibits promising biosafety and therapeutic efficacy against hvKp-induced pulmonary infections and dissemination in a murine model. These findings suggest that phage PK2420 may be a potential option for the clinical treatment of hvKp infections.

## INTRODUCTION

*Klebsiella pneumoniae* is an epidemic opportunistic pathogen capable of colonizing various sites in the human body, including the axilla, intestinal tract, and nasopharynx (1). It is classified into hypervirulent *K. pneumoniae* (hvKp) and classic *K. pneumoniae* based on virulence genes and associated infectious diseases (2). The first known case of hvKp was identified in Taiwan in 1986 (3); since then, it has achieved global distribution (4). Compared with classic *K. pneumoniae*, hvKp exhibits substantially higher bacterial virulence and harbors unique virulence genes, such as *iroB*, *iucA*, *peg-344*, *rmpA*, and *rmpA2* (5). HvKp is commonly characterized by a hypermucoviscosity phenotype, identified by the string test where a single colony is lifted with an inoculating loop. A positive result is indicated by a string length of ≥5 mm. This phenotype is attributed to the presence of two capsular polysaccharide regulator genes (*rmpA* and *rmpA2*) and multiple siderophore gene clusters (6, 7). Unlike classic *K. pneumoniae*, hvKp can infect healthy individuals in the community and is associated with poor prognoses and high mortality rates. HvKp often causes infections at multiple sites or spreads from a primary site, leading to invasive infections such as liver abscesses, pneumonia, endophthalmitis, and central nervous system infections (2).

The most common serotypes of hvKp include K1, K2, K5, K20, K54, and K57. Among these, K20 is one of the most prevalent after K1 and K2 (8). hvKp K20 is often associated with nosocomial outbreaks and serves as a dominant epidemic strain in certain regions. In Wenzhou, China, approximately 33% of hypermucoviscous carbapenem-resistant *K. pneumoniae* isolates exhibited the K20 serotype (9); in Tehran, Iran, over 55% of hvKp isolates reportedly displayed the K20 serotype (10, 11). According to the China Antimicrobial Resistance Surveillance System (CHINET), the proportion of *K. pneumoniae* among pathogenic bacteria nationwide increased from 14.39% to 15.17% between 2018 and 2022 (12, 13). Meanwhile, carbapenem resistance in *K. pneumoniae* rose from 7.6% to 10% between 2015 and 2022 (14).

While the acquisition of genes aligns with evolutionary processes, environmental adaptation may play a more transient role, focusing on phenotypic changes rather than permanent genetic alterations. Additionally, external factors such as globalization and travel have indirectly influenced the evolutionary process by facilitating the spread of these strains. As a result, multidrug-resistant hvKp has emerged and rapidly increased in recent years (15). In China, the emergence of the multidrug-resistant phenotype of hvKp has made traditional antibiotic therapies, such as carbapenems, less effective (16). Furthermore, conventional antibiotic treatments such as ceftriaxone for community-acquired pneumonia treatment lack specificity, targeting both pathogenic bacteria and beneficial members of the gut microbiota, which can lead to disruptions in the microbiome and potential adverse effects (17). Thus, there is an urgent need to develop new antibacterial approaches that are not only highly specific, but also safe and effective.

The use of bacteriophages to treat bacterial infections dates back more than 100 years (18). As natural predators of bacteria, bacteriophages have been widely utilized to treat infections at various sites, including the lungs and skin (19), and against numerous pathogenic bacteria such as *Salmonella*, *Bacillus anthracis*, *Staphylococcus*, and *K. pneumoniae* (20). The antibacterial mechanism of phages is distinct from that of antibiotics, characterized by high host specificity, enabling them to precisely target specific multidrug-resistant bacteria (21). Compared to conventional drug development, it is relatively inexpensive to isolate, characterize, and propagate phages, especially in Kenya, Tanzania, and other developing countries (22). Moreover, phage therapy provides the benefit of safety (23). In recent years, phages targeting hvKp have been reported, primarily focusing on strains with K1 and K2 serotypes (24–26). However, these studies were limited to investigations of phage biology and genomic characteristics (24, 27) or lacked detailed explorations into therapeutic mechanisms *in vivo* (25). Research on phages targeting hvKp K20 is particularly scarce and has been restricted to studies of depolymerases (27). Further analyses regarding the therapeutic mechanisms and pharmacology of phages are needed (18).

In this study, a novel lytic phage, PK2420, with high specificity for hvKp, was isolated from hospital sewage. Comprehensive analyses of its morphological, biological, and genomic characteristics were conducted. Phage PK2420 effectively lysed *K. pneumoniae*, particularly the K20 serotype; it also demonstrated high efficacy and safety in terms of mitigating inflammation and maintaining normal body temperature within an hvKp-induced murine pneumonia model. These findings indicate that PK2420 has potential as a novel treatment for multidrug-resistant hvKp infections in clinical settings.

## MATERIALS AND METHODS

### Bacterial strains, cells, culture conditions, and mice

A total of 82 *K*. *pneumoniae* strains were clinically isolated from patients with various conditions, including 32 strains from pneumonia patients, 46 strains from pyogenic liver abscess patients, 3 strains from auto-brewery syndrome patients, and 1 strain from a non-alcoholic steatohepatitis and auto-brewery syndrome patient. These strains were previously stored at −80°C in our laboratory (28, 29). Whole-genome sequencing was performed on all strains. Multilocus sequence typing (MLST) was conducted using the *K. pneumoniae* MLST database from the Pasteur Institute (https://bigsdb.pasteur.fr/klebsiella/), based on the housekeeping genes *gapA*, *infB*, *mdh*, *pgi*, *phoE*, *rpoB*, and *tonB* (30). Serotyping was confirmed using the same database (31) and the Kaptive tool (32), according to analyses of capsular polysaccharide cluster genes. Detailed strain information is provided in Table S1. The string test was performed as previously described (5). Bacteria and phage were grown in yeast-extract-peptone-dextrose (YPD) or Luria-Bertani (LB) media. YPD broth consisted of 10 g/L yeast extract, 20 g/L peptone, and 20 g/L glucose; LB broth contained 10 g/L tryptone, 5 g/L yeast extract, and 10 g/L NaCl. A multidrug-resistant hvKp strain, K2420, served as the host for phage isolation and proliferation. Human lung carcinoma A549 cells were cultured in PMI-1640 medium supplemented with 10% fetal calf serum (Gibco, USA) at 37°C with 5% CO_2_.

Specific-pathogen-free male C57BL/6J mice (23–25 g) were purchased from Beijing Vital River Laboratory Animal Technology Co., Ltd. (Beijing, China).

### Analysis of antibiotic resistance genes and virulence factors

Antibiotic resistance genes in bacterial strains were analyzed using the Comprehensive Antibiotic Research Database (CARD), which includes extensive data regarding pathogens, chromosomes, genomic islands, plasmids, whole-genome sequence assemblies, and alleles for resistome predictions. The Resistance Gene Identifier tool in CARD was utilized for sequence alignment to identify putative antibiotic resistance genes, based on a blastp *e* value threshold of ≤1e−30 (33). Virulence factor analysis was performed using the Virulence Factor Database (VFDB), which contains 11,822 virulence factors from 74 bacterial pathogens, including *K. pneumoniae* (34).

### Isolation, purification, and observation of phage PK2420

A total of 50 mL untreated sewage samples was obtained from the Capital Institute of Pediatrics in Beijing, China. The samples were centrifuged at 5,000 × *g* for 20 min, and the supernatant was filtered through a 0.22 µm membrane. The filtrate was mixed with an overnight culture of *K. pneumoniae* K2420 grown in an LB medium to detect the presence of the phage (35). A double agar overlay plaque assay (36) was used to isolate the phage, with repeated purification steps to ensure phage purity. Additionally, the double agar overlay plaque assay was also utilized to quantify phage titers. Transmission electron microscopy was conducted at 80 kV to observe the morphological features of the isolated phage.

### Determination of optimal multiplicity of infection (MOI)

Tenfold serial dilutions of phage, ranging from 10^9^ to 10^3^ plaque-forming units (PFU)/mL, were mixed with *K. pneumoniae* K2420 host bacteria (10^7^ colony-forming units [CFU]/mL) in log-phase growth. This approach corresponded to MOIs ranging from 10^2^ to 10^−4^. The mixtures were incubated overnight at 37°C in a shaker. A double agar overlay plaque assay was performed to measure phage quantities. The MOI resulting in the highest phage yield was considered optimal.

### One-step growth curve

A one-step growth curve was used to analyze phage replication kinetics (37). The phage and host strain were mixed at the optimal MOI and incubated in a shaker at 37°C for 160 min. Phage titer was measured every 10 min using the double agar overlay plaque assay. Burst size was calculated as the ratio of the number of phages released during the first amplification to the initial number of infected bacterial cells in the latency phase (38).

### Phage stability according to temperature and pH

Phage stability was assessed by incubation at various temperatures (0, 4, 10, 20, 30, 40, 50, 60, 70, 80, 90, and 100°C) and at pH levels ranging from 3 to 13 for 1 h at 37°C. Phage titer was determined using the double agar overlay plaque assay.

### Host range determination

Phage host range was evaluated using 82 clinical *K. pneumoniae* isolates exhibiting different sequence types and serotypes. A standard spot test and efficiency of plating (EOP) assay were performed as previously described (39). Bacteria were considered sensitive to the phage if clear, transparent plaques formed on the plates. The EOP for the K2420 strain was regarded as 100% control.

### *In vitro* lysis assay

*In vitro* lysis assay was performed as described in the previous study with slight changes (25). To evaluate phage lytic ability, host strain cultures at 10^7^ CFU/mL in log-phase growth were mixed with phage at MOIs ranging from 10^2^ to 10^−6^, and then added into 96-well plates. Host strain culture without phage and LB medium was used as a control and blank control, respectively. The cultures were incubated at 37°C, and optical density at 600 nm (OD_600_) was measured every 15 min.

### Biofilm inhibition and elimination assays

The crystal violet staining method was used to quantify biofilms (40). To assess phage ability to inhibit biofilm formation, log-phase host strain cultures at 10^7^ CFU/mL were mixed with phage at MOIs ranging from 10^2^ to 10^−6^ in YPD broth, then incubated in 96-well plates at 37°C for 48 h. A control group consisting of host strain cultures mixed with YPD broth was used to assess biofilm formation without phage treatment. After incubation, the culture medium was removed, and the wells were washed three times with normal saline. Biofilms were subsequently fixed with 2.5% glutaraldehyde solution for 30 min at 37°C. Staining with 0.75% crystal violet and decolorization with 95% ethanol were performed for 30 min each. Finally, the optical density at 570 nm (OD_570_) was measured to quantify biofilm levels.

To evaluate the phage ability to eradicate preformed biofilms, log-phase host strain cultures at 10^7^ CFU/mL were incubated in YPD broth at 37°C for 48 h within 96-well plates to establish biofilms. The supernatants were removed, and the remaining bacteria were washed out three times with normal saline. Next, phage dilutions of high (10^9^ PFU/mL), medium (10^6^ PFU/mL), and low (10^3^ PFU/mL) concentrations, as well as YPD broth (control), were added and incubated for 24 h. Washing, fixation, staining, decolorization, and measurement steps were performed as described above.

### DNA extraction and genome sequence analysis

Phage genomic DNA was extracted using the phenol-chloroform method, as previously described (41). Whole-genome sequencing was performed on the Illumina NovaSeq 6000 platform to provide an overview of the genetic content. Potential coding sequences were identified using PHASTER (42), and putative homologies with predicted phage proteins were identified via blastp. Genome assembly, annotation, and visualization were conducted using Proksee (https://proksee.ca/) (43). Phylogenetic trees were constructed based on amino acid sequences of the DNA polymerase using MEGA 11.0 with the maximum likelihood method and 1,000 bootstrap replications. Virulence factor and antibiotic resistance gene analyses were conducted using VFDB (34) and Resfinder 4.0 (44), respectively.

### Assessment of phage cytotoxicity

Phage cytotoxicity in human lung carcinoma A549 cells was evaluated using a commercial cell counting kit-8 (CCK-8) (Dojindo, Shanghai, China), in accordance with the manufacturer’s instructions. A549 cells (1 × 10^4^ cells/well) were seeded in 96-well plates and incubated for 24 h to allow adherence. After supernatant removal, 10-fold serial dilutions of the phage (ranging from 10^1^ to 10^9^ PFU/mL) were added and incubated at 37°C with 5% CO_2_ for 72 h. A549 cells without phage treatment served as the control. After incubation with the phage, CCK-8 reagent was added to each well and incubated for 1 h. Optical density at 450 nm (OD_450_) was measured to calculate cell viability based on the instructions of the assay kit.

### HvKp-induced pneumonia and phage PK2420 therapy in a mouse model

We performed a preliminary experiment to establish an animal model based on the approach utilized in previous studies (35, 45). Each mouse received an intratracheal injection of *K. pneumoniae* K2420 at doses ranging from 10^2^ to 10^8^ CFU, and the 7-day mortality rate was monitored (Fig. S1A). To study the quantitative dynamics and interactions between the phage and bacteria, as well as other infection-related characteristics, we selected a dose of 6.6 × 10^3^ CFU per mouse to create a pneumonia model. This dose resulted in a high mortality rate over 7 days while avoiding rapid death.

All mice were divided into four groups, anesthetized, and administered intratracheal injections as follows: (i) the K2420 group (infection group, *n* = 41) received 6.6 × 10^3^ CFU of *K. pneumoniae* K2420, followed by normal saline 2 h later; (ii) the K2420 + PK2420 group (treatment group, *n* = 36) received 6.6 × 10^3^ CFU of *K. pneumoniae* K2420, followed by 2 × 10^9^ PFU of phage PK2420 2 h later; (iii) the phage group (safety group, *n* = 36) received normal saline, followed by 2 × 10^9^ PFU of phage PK2420 2 h later; and (iv) the control group (*n* = 36) received normal saline, followed by another normal saline dose 2 h later. Five mice from each group were euthanized and dissected at 2, 6, 12, 24, 48, and 72 h post-infection. Lung tissue samples were homogenized to monitor bacterial loads, phage concentrations, and cytokine levels, including interleukin-1β (IL-1β), IL-6, and tumor necrosis factor-alpha (TNF-α); these measurements enabled the assessment of inflammation. To assess phage susceptibility, various concentrations of PK2420 were applied to lawns of residual *K. pneumoniae* isolates from lung tissue. Lung, liver, spleen, and kidney tissues were fixed with 4% paraformaldehyde and subjected to hematoxylin-eosin staining at 24, 48, and 72 h, as well as 7 days, to study pathological changes. To confirm the safety and efficacy of phage therapy, 6 mice per group (11 mice in the infection group due to expected mortality in this group) were included in the survival analysis. Body temperature was measured every 24 h for 7 days. After the survival analysis period, all surviving mice were euthanized and dissected; tissue samples were analyzed as described above.

### Histopathological scoring of lung tissues

Lung injury in each group was evaluated using the following standardized scoring system for lung tissue pathology: grade 0 = no inflammatory response in the alveolar walls; grade 1 = diffuse inflammatory reaction, predominantly neutrophilic, in the alveolar walls without thickening; grade 2 = widespread infiltration of neutrophilic and mononuclear inflammatory cells in the alveolar walls, accompanied by mild thickening; grade 3 = obvious thickening (two- to threefold) of the alveolar walls due to inflammatory cell presence; grade 4 = substantial thickening of the alveolar walls, with consolidation affecting up to 25% of lung tissue; and grade 5 = extensive thickening of the alveolar walls, resulting in consolidation that involved more than 50% of lung tissue (46).

### Statistical analysis

Data are presented as mean ± standard deviation. Comparisons among multiple groups were conducted using one-way analysis of variance, with post hoc analysis employing least significant difference when variances were homogeneous and Tamhane’s T2 test when variances were heterogeneous. Survival analysis was calculated using the Kaplan–Meier method. Statistical analyses were conducted using SPSS 20.0 software (IBM Corp., Armonk, NY, USA). Differences were considered statistically significant at *P* values of <0.05 (*), 0.01 (**), or 0.001 (***).

## RESULTS

### *K. pneumoniae* K2420 carries virulence factors, antibiotic resistance genes, and the ability to form biofilms

*K. pneumoniae* K2420 was clinically isolated from a nasopharyngeal swab sample. Sequence analysis identified it as an ST268, K20 serotype strain, carrying virulence genes such as *rmpA*, *rmpA2*, and others (listed in Table S1). The minimum lethal dose of K2420 in mice, administered by intratracheal injection, was 500 CFU (Fig. S1A). Consistent with its virulence factor profile, K2420 was classified as an hvKp strain. K2420 exhibited a hypermucoviscosity phenotype, confirmed by a positive string test result (Fig. S1B); it formed prominent biofilms in the YPD medium after 48 h (Fig. S1C). Metagenomic analysis using CARD identified 36 resistance genes, including *baeR*, *KpnG*, and *msbA*. These genes were associated with resistance to 43 antibiotics, including amikacin, ciprofloxacin, azithromycin, tetracycline, cefotaxime, and metronidazole, among others (Fig. S2; Table S2). Based on this profile, K2420 was categorized as a multidrug-resistant *K. pneumoniae* strain.

### Isolation of phage PK2420, which targets hvKp (K20 serotype)

**Fig 1:**
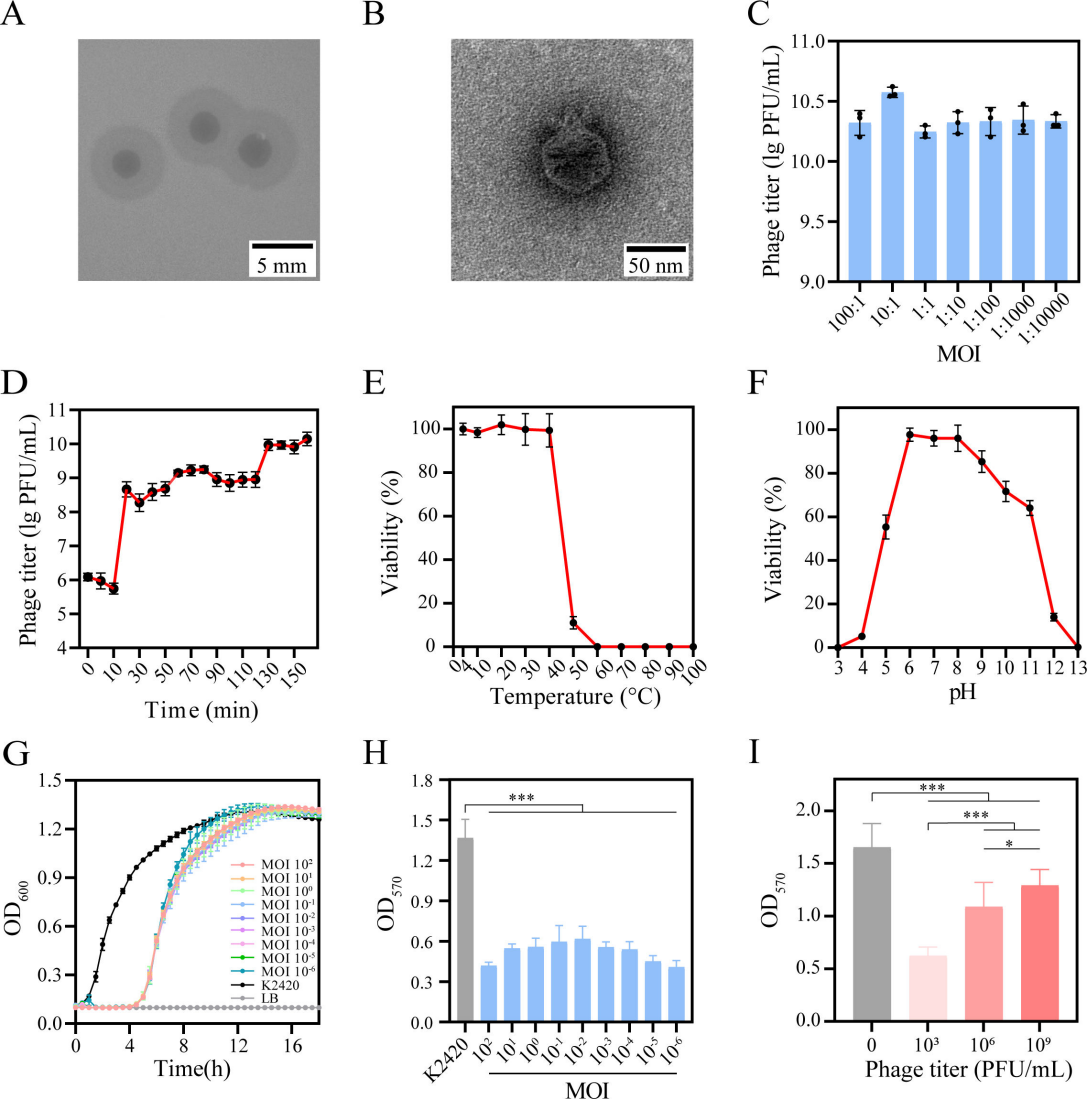
Comprehensive analysis of phage PK2420, including morphology, biology, and biofilm impact (**A**) Visualization of phage PK2420 plaques on double-layer agar plates. (**B**) Transmission electron microscopy image of PK2420. (**C**) Determination of the optimal multiplicity of infection (MOI) for PK2420. (**D**) One-step growth curve for PK2420. (**E**) Assessment of the thermal stability of PK2420. (**F**) Evaluation of the pH stability of PK2420. (**G**) *In vitro* bacteriophage inhibition assay. (**H**) Inhibition of biofilm formation by PK2420 *in vitro*. (**I**) Efficacy of PK2420 in eradicating pre-formed biofilms. Data are presented as mean ± standard deviation (SD) from three independent experiments (*n* = 3).

A lytic phage named PK2420 (GenBank accession number PQ589833) was isolated from hospital sewage using K2420 as the host strain. The phage formed clear plaques with halos on double-layer agar plates (average diameter: 6.26 ± 0.20 mm; Fig. 1A), demonstrating a strong lytic effect. Transmission electron microscopy revealed that PK2420 exhibits an icosahedral head approximately 55 nm in diameter, along with a short tail (Fig. 1B). Eighty-two *K. pneumoniae* isolates, representing 24 K types and 31 K locus types (listed in Table S3), were used to determine the host range of PK2420. The phage also lysed *K. pneumoniae* strain 16101 (ST420, K20), suggesting that it shows specificity for K20 serotype strains. However, EOP assays indicated that the lytic efficiency of PK2420 was lower against K16101 than against K2420 (Fig. S1D).

### Phage PK2420 effectively lyses K2420 and remains stable *in vitro*

To further elucidate the characteristics of phage PK2420, the optimal MOI was detected as 10 (Fig. 1C). One-step growth curve analysis revealed an initial latency phase of 10 min, followed by a rapid rise over the next 20 min and a plateau lasting approximately 70 min, with an average burst size of 37.4 PFU per cell (Fig. 1D); these findings demonstrated that PK2420 can quickly and efficiently kill K2420. The phage remained stable at temperatures ranging from 0 to 40°C and pH values between 6 and 9 (Fig. 1E and F), indicating its suitability for use in physiological conditions. Additionally, PK2420 completely inhibited the growth of its host strain at MOIs ranging from 10^2^ to 10^−6^ for at least 4 h (Fig. 1G), demonstrating strong lytic activity *in vitro*.

### Phage PK2420 inhibits and removes K2420 biofilms

Biofilms are complex bacterial structures composed of extracellular polymeric substances secreted by bacteria. These structures enhance bacterial resistance to antimicrobial agents and allow evasion of the host immune response. The results demonstrated that PK2420 substantially suppressed biofilm formation by K2420, with inhibition rates ranging from 54% to 70% at MOIs of 10^2^ to 10^−6^ over a 48-h period (Fig. 1H). Additionally, when applied to pre-formed biofilms, PK2420 effectively reduced biofilm mass by 28–62% within 24 h(Fig. 1I). These findings indicated that PK2420 can inhibit biofilm formation and eradicate pre-existing biofilms.

### Genomic features and protein predictions of PK2420

**Fig 2:**
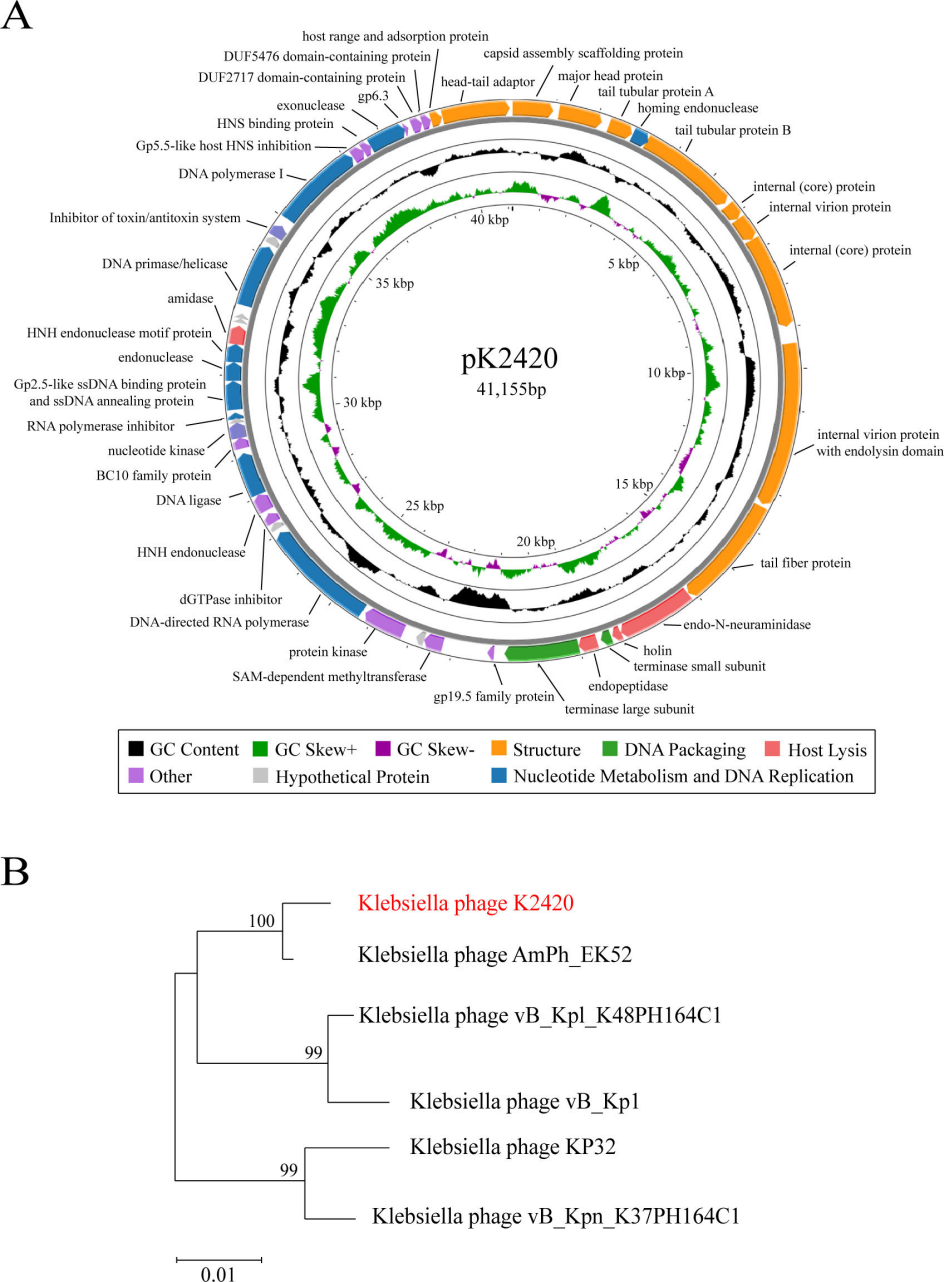
Genomic features of PK2420. (**A**) Genomic map of PK2420. (**B**) Phylogenetic analysis of PK2420 based on DNA polymerase gene sequences, constructed using the maximum likelihood method. Bootstrap values are shown at the nodes to indicate the robustness of phylogenetic relationships. Scale bars represent amino acid substitutions per site.

The genome of phage PK2420 is 41,155 bp long, with a GC content of 53% and 46 predicted coding sequences (Fig. 2A). Comparative genome analysis revealed that PK2420 shares the highest similarity with *Klebsiella* phage phi1_146027 (accession PP889477.1)—97.60% identity and 94% query coverage (Table S4)—confirming the novelty of PK2420. Phylogenetic analysis based on DNA polymerase sequences showed that PK2420 belongs to the phylum *Uroviricota*, class *Caudoviricetes*, family *Autographiviridae*, and genus *Przondovirus*, consistent with its morphological characteristics (Fig. 1B and 2B). Predicted proteins included 2 DNA packaging proteins, 5 host lysis proteins, 11 structural proteins, 11 nucleotide metabolism and DNA replication proteins, 11 additional functional proteins, and 6 hypothetical proteins (Fig. 2A; Table S5). Notably, the genomic analysis revealed that PK2420 does not carry antibiotic resistance, virulence, or lysogenic genes (Fig. 2A; Table S5). Thus, this novel phage exhibits a robust safety profile and has high therapeutic potential.

### Phage PK2420 demonstrates biosafety *in vitro* and *in vivo*

**Fig 3:**
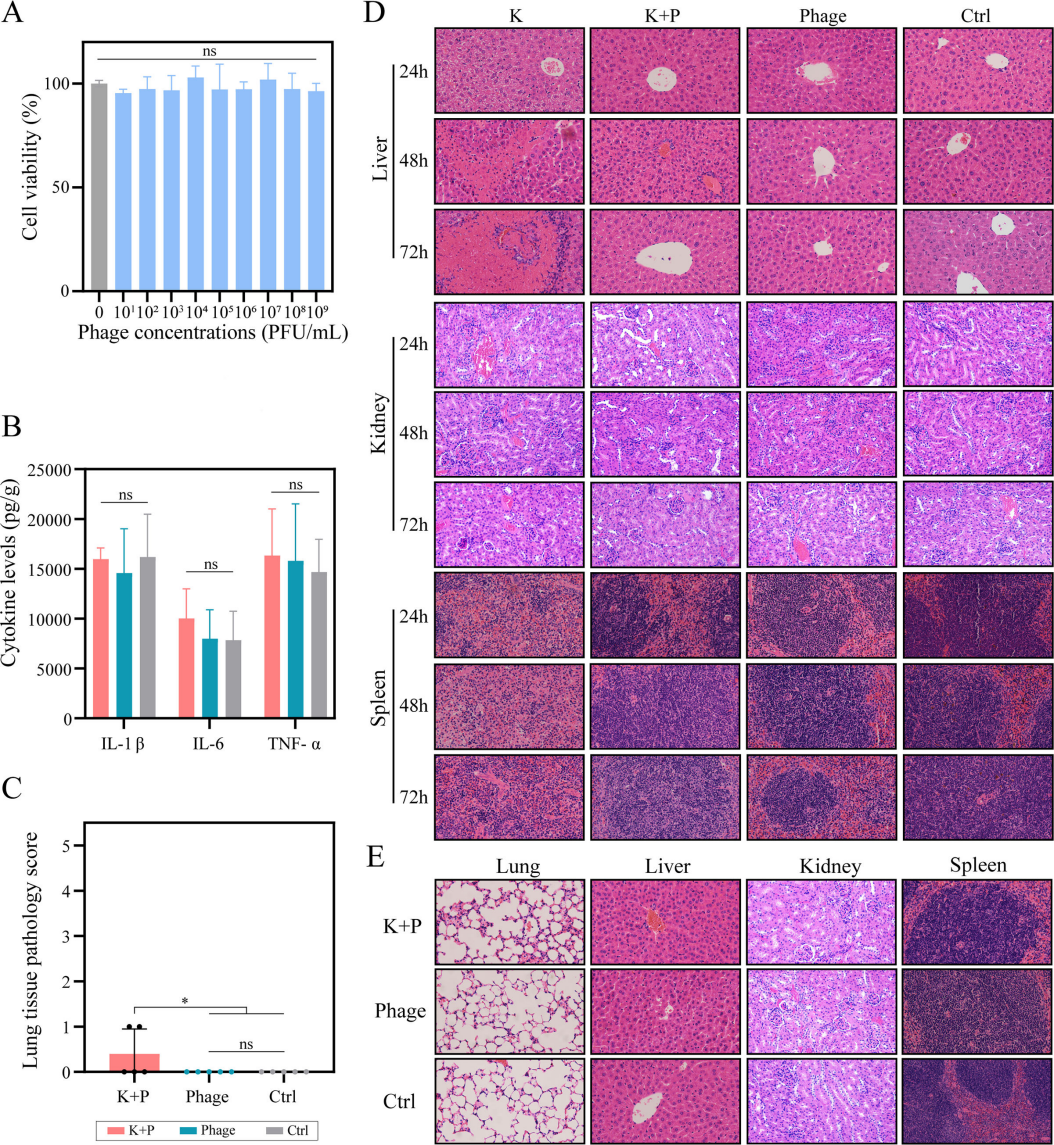
Safety assessment of phage therapy *in vitro* and *in vivo*. (**A**) Evaluation of phage cytotoxicity using a cell proliferation assay. Data are presented as mean ± SD from three independent experiments (*n* = 3). (**B**) Levels of proinflammatory cytokines IL-1β, IL-6, and TNF-α at 168 h post-infection in treatment, phage, and control groups. (**C**) Histopathological scores of lung tissue sections at 168 h post-infection. Scoring criteria are described in the Materials and Methods section. Values are expressed as mean ± SD (*n* = 5 mice/group). *P < 0.05 (one-way ANOVA). (**D**) Histopathological analysis of liver, kidney, and spleen tissues (hematoxylin and eosin stain, ×200) at 24, 48, and 72 h post-infection. (**E**) Histopathological analysis of lung, liver, kidney, and spleen tissues (hematoxylin and eosin stain, ×200) at 168 h post-infection. K and K + P refer to infection and treatment control groups, respectively. ANOVA, analysis of variance; IL-1β, interleukin-1 beta; SD, standard deviation; TNF-α, tumor necrosis factor-alpha.

**Fig 4:**
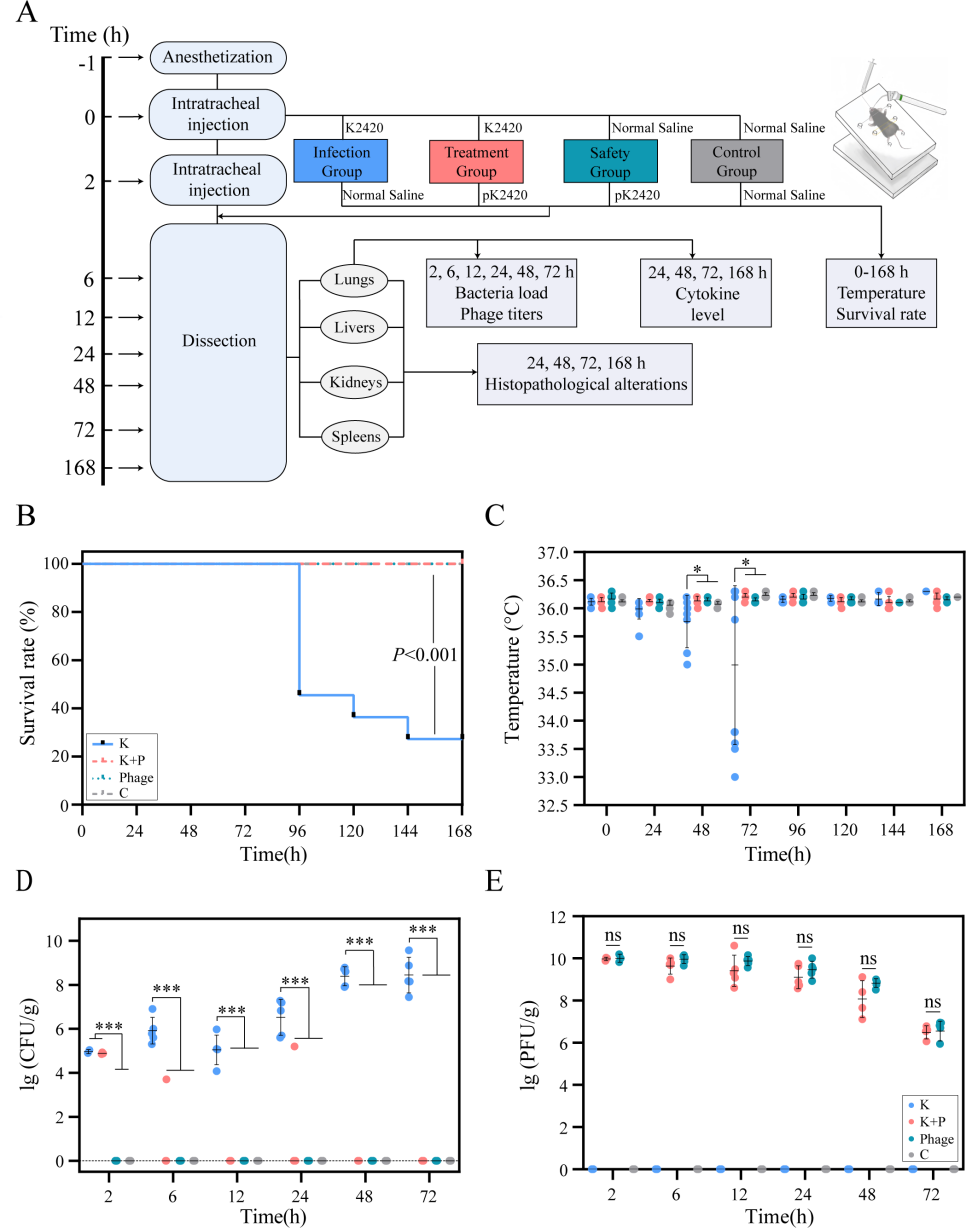
Phage PK2420 improves survival rate, maintains body temperature, and reduces bacterial loads in a murine model of hvKp-induced pneumonia. (**A**) Schematic representation of the experimental workflow for phage therapy. (**B**) Kaplan–Meier survival curves for *K. pneumoniae*-infected mice after phage therapy. (**C**) Body temperature fluctuations in mice over 168 h. (**D**) Quantitative assessment of bacterial loads in mice over a 72-h period. (**E**) Phage titers in mice over a 72-h period. K and K + P refer to infection and treatment control groups, respectively. Values are expressed as mean ± SD (*n* = 5 mice/group). *P < 0.05; ***P < 0.001 (one-way ANOVA). ANOVA, analysis of variance; hvKp, hypervirulent *Klebsiella pneumoniae*; SD, standard deviation.

To assess the safety of phage therapy *in vitro*, a cell proliferation assay was performed. After 72 h of incubation with PK2420, no significant difference in cell viability was observed between the phage and the control groups (Fig. 3A), indicating that PK2420 does not exhibit cytotoxicity toward A549 cells *in vitro. In vivo*, biosafety was assessed by several indicators, including survival rates, body temperature, cytokine levels, histopathological findings, and other relevant factors. The survival rates of the treatment and phage groups remained at 100% over 7 days, identical to the control group (Fig. 4B). Mice in the treatment and phage groups maintained a normal body temperature of approximately 36°C (Fig. 4C) and showed no apparent clinical symptoms, such as rattling breathing noises, hunched posture, listlessness, and matted or puffed-up fur. Cytokine levels and pathological examinations of lung, liver, and kidney tissues revealed no significant differences between the phage and the control groups after infection (Fig. 3B through E). Histopathological analysis of lung tissues in the treatment group showed a mild diffuse inflammatory reaction, predominantly neutrophilic, in the alveolar walls without any associated thickening (Fig. 3C and E). Mild hyperplasia of marginal zone macrophages within the spleen was observed in the phage and treatment groups, likely reflecting macrophage involvement in phage particle clearance (Fig. 3D and E). These results indicated that PK2420 does not cause death or induce significant inflammatory responses or toxicity in lung, kidney, liver, or spleen tissues *in vivo*. Overall, PK2420 exhibited favorable safety profiles *in vitro* and *in vivo*, supporting its potential as a therapeutic agent for *K. pneumoniae* infections.

### Phage PK2420 improves survival rate and reduces K2420 load *in vivo*

Preliminary experiments demonstrated that as few as 500 CFU of *K. pneumoniae* K2420 administered via intratracheal injection could cause mortality in mice (Fig. S1A). In the main experiment, we found that 6.6 × 10^3^ CFU of K2420 caused a 72% mortality rate between 72 h and 7 days post-infection. This model was used to evaluate the therapeutic effect of PK2420 *in vivo* (Fig. 4A). Survival curve analysis showed a 100% survival rate in the treatment group, indicating that intratracheal administration of PK2420 effectively prevented mortality (Fig. 4B). Whereas mice in the control, phage, and treatment groups maintained normal body temperature and physiological status, mice in the infection group exhibited lethargy, reduced activity, and significant hypothermia between 48 and 72 h post-infection (Fig. 4C). Body temperature data from the infection group after 72 h were excluded from analysis because the deaths of some mice might have interfered with statistical accuracy.

At 2 h post-injection, bacterial loads in the infection and treatment groups confirmed the successful establishment of the pneumonia model. In the infection group, bacterial concentrations increased nearly 10,000-fold within 72 h post-infection, stabilizing at 10^7^–10^8^ CFU/g. In contrast, in the treatment group, K2420 was completely cleared by the phage within 72 h, except in one of five mice at 6 and 24 h, where transient bacterial residues were observed. Both isolates displayed resistance to PK2420 but did not proliferate further (Fig. 4D; Fig. S3; Table S6). Phage concentrations in the treatment and phage groups at 2 h post-injection confirmed effective delivery of PK2420 to the lungs via intratracheal injection. Phage titers stabilized at 10^9^–10^10^ PFU/g within 24 h and gradually declined to approximately 10^6^ PFU/g by 72 h, with no significant differences between the two groups (Fig. 4E).

### Phage PK2420 effectively alleviates hvKp-induced damage to the lung, liver, and spleen

**Fig 5:**
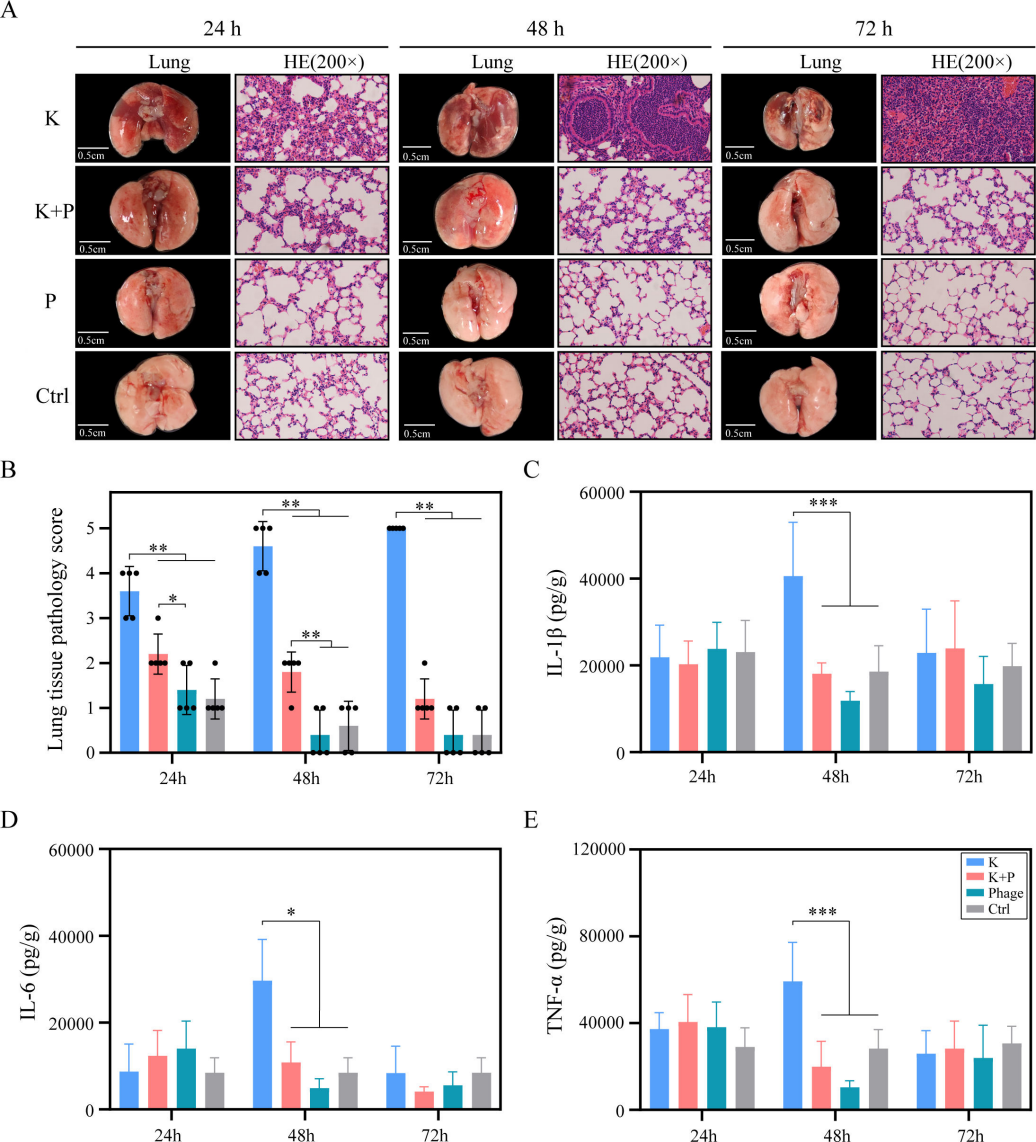
Phage PK2420 alleviates hvKp-induced pneumonia in a murine model. (**A**) Histopathological analysis of lung tissues (hematoxylin and eosin stain, ×200) at 24, 48, and 72 h post-infection. (**B**) Histopathological scores of lung tissue sections. Scoring criteria are based on the degree of alveolar wall thickening and the presence of inflammatory cells, as described in Materials and Methods. (C–E) Levels of proinflammatory cytokines IL-1β, IL-6, and TNF-α at 24, 48, and 72 h post-infection. K and K + P refer to infection and treatment control groups, respectively. Values are expressed as mean ± SD (*n* = 5 mice/group). *P < 0.05; **P < 0.01 (one-way ANOVA). ANOVA, analysis of variance; hvKp, hypervirulent *Klebsiella pneumoniae*; IL-1β, interleukin-1 beta; SD, standard deviation; TNF-α, tumor necrosis factor-alpha.

*K. pneumoniae* K2420 infection caused pulmonary, hepatic, and splenic damage. In the lungs, this infection led to congestion, expansion, and necrosis. Histopathological examination at 24 h post-infection revealed pronounced inflammatory cell infiltration in the alveolar walls; some areas showed three to five fold thickening (Fig. 5A). By 48 h, pulmonary abscesses had formed (Fig. 5A), accompanied by significantly elevated cytokine levels, including IL-1β, IL-6, and TNF-α (Fig. 5C through E). At 72 h, the abscesses had coalesced, affecting more than 50% of lung tissue (Fig. 5A). Lung injury severity progressively increased from 24 to 72 h, as indicated by rising lung pathology scores (Fig. 5B). Additionally, K2420-induced hepatic damage was evident, beginning with eosinophilic degeneration of hepatocytes at 24 h. This degeneration progressed to bridging necrosis and increased inflammatory cell infiltration at 48 h, then culminated in extensive necrosis, hemorrhage, and dense inflammatory cell infiltration within portions of the hepatic lobules at 72 h (Fig. 3D). In the spleen, K2420 infection disrupted the architecture of splenic corpuscles, caused the disappearance of germinal centers, and led to partial restructuring of splenic corpuscles. Pathological findings also showed diffuse lymphocytic hyperplasia, multinucleated giant cell proliferation, and granuloma formation. These changes became increasingly severe from 0 to 72 h post-infection (Fig. 3D). K2420 did not induce renal damage; no significant differences in nephropathological findings were observed among the four groups (Fig. 3D).

In the treatment group, lung tissues exhibited less thickening of the alveolar walls, and the alveolar structure remained intact. Lung pathology scores and cytokine levels were significantly lower in the treatment group than in the infection group. Furthermore, lung injury showed progressive recovery between 24 and 72 h in the treatment group; injury scores became indistinguishable from those of the phage and control groups by 72 h (Fig. 5A and B). The liver displayed only mild eosinophilic changes in some areas. In the spleen, corpuscles exhibited hyperplasia, with active germinal center proliferation, increased marginal zone macrophages, and dilated, congested splenic sinusoids, thereby reflecting the immune response to infection (Fig. 3D). These findings indicated that PK2420 significantly mitigated pulmonary, hepatic, and splenic injuries.

## DISCUSSION

HvKp was first identified in seven cases of community-acquired pyogenic liver abscesses in Taiwan (3). Since then, hvKp has gradually emerged worldwide, evolving into a multidrug-resistant pathogen (2). Thus, there is a critical need for alternative therapies to treat hvKp in clinical settings. Considering its unique advantages, phage therapy has been applied to several diseases and constitutes a promising complementary approach to conventional antibiotics.

In this study, we successfully isolated a bacteriophage, PK2420, which targets hvKp (K20 serotype). Comprehensive *in vitro* and *in vivo* analyses demonstrated its biological and kinetic properties, favorable biosafety profile, and therapeutic efficacy. In terms of phage biosafety, sequence analysis confirmed the absence of genes associated with antibiotic resistance, virulence, or lysogeny. *In vitro* experiments showed no cytotoxic effects. *In vivo* treatment outcomes were highly favorable, with a 100% survival rate and stable body temperatures in treated mice throughout the study. Histopathological examinations of the lungs, liver, and kidneys, along with measurements of lung inflammatory cytokine levels, remained within normal ranges at 24, 48, 72, and 168 h. A slight increase in splenic phagocytic cells was observed, which likely reflected splenic macrophage-mediated clearance of bacteriophages and suggested an immune-related response (47). However, further research is needed to fully elucidate the interactions between bacteriophages and host immune mechanisms. Consistent with a previous report (23), no severe side effects were observed after PK2420 administration, confirming its excellent biosafety profile.

Regarding therapeutic efficacy, phage PK2420 exhibited a short latent period, an appropriate burst size, and an optimal MOI, enabling rapid and specific lysis of hvKp. It significantly inhibited biofilm formation and cleared pre-formed biofilms. The phage remained stable under physiological temperature and pH conditions. *In vivo* treatment with PK2420 improved survival rates, maintained normal body temperatures, and substantially reduced pulmonary inflammation and hepatosplenic damage caused by hvKp. Additionally, PK2420 effectively reduced inflammatory cytokine levels, preventing cytokine storm onset and demonstrating robust therapeutic effects.

In our previous study regarding *Klebsiella aerogenes* phage (48), the treatment group exhibited *in vivo* bacteriophage amplification, with significantly higher phage titers relative to mice that only underwent phage delivery. However, in the present study, no significant differences in phage titer were observed between the treatment and phage-only groups. PK2420 rapidly cleared bacteria in the lungs, but no increase in phage titer occurred, presumably because the host bacterial concentration was less than one-millionth of the bacteriophage titer; this dilution rendered the bacterial abundance insufficient for phage replication. Furthermore, the clearance rates of different phage types and titers *in vivo* may vary (49), but the underlying mechanisms require further investigation. Although PK2420 significantly reduced bacterial loads in the lungs, two residual *K. pneumoniae* isolates were detected at 6 and 24 h post-infection. However, no residual bacteria were observed at 12, 48, or 72 h, or at 7 days post-infection. These findings suggest that resistant bacteria represented only a very low proportion (approximately 5%) of samples from the treatment group and were only transiently present within the first 24 h of treatment. Similar transient emergence of resistant strains has been reported during phage therapy for *K. pneumoniae* serotypes K1 and K2, possibly due to mutations in the *wzc* and *wcaj* genes (50). Additionally, the transient nature of resistance might be linked to the selective pressure exerted by the phage. Once the selective pressure is removed, resistant strains may revert to their wild-type phenotype due to the high metabolic cost associated with maintaining these mutations. Further genomic and phenotypic analyses are needed to elucidate the specific roles of *wzc* and *wcaj* in phage resistance and to assess whether these mutations compromise other bacterial functions, such as immune evasion or colonization efficiency. A previous study showed that even without complete bacterial clearance, phage therapy can reduce *K. pneumoniae* virulence and enable the host immune system to achieve clearance (51). Moreover, phage-resistant *K. pneumoniae* strains have been shown to lose multidrug resistance genes (52). Based on these findings, we speculate that the two resistant strains identified in this study were transient and infrequent; they did not affect the overall efficacy of PK2420-based phage therapy.

Nevertheless, this study has several limitations that can be broadly categorized into the biological characteristics of phages and the therapeutic features of phage therapy. For limitations of biological characteristics of phage, only two K20 serotype strains of hvKp were investigated, leaving it unclear whether phage PK2420 can lyse all K20 strains. The mechanisms underlying phage resistance in the K2420 strain were not studied and merit further exploration. The study noted immune responses (e.g., macrophage activation), but the broader implications of immune modulation, including the potential for unintended immune effects, were not fully explored. The study reported stable phage titers *in vivo*, but the dynamics of phage clearance and the long-term impact on the host microbiome were not assessed. For limitations of therapeutic features of phage therapy, the phages were only used for one time point, thus optimal treatment timing and duration require more comprehensive investigation. The study lacked comparisons with standard antibiotic treatments or phage-antibiotic combinations, which are critical to contextualizing therapeutic efficacy and could address the transient resistant strains. Long-term effects of phage therapy, including potential for recurrence of infection, microbial dysbiosis, or immune sensitization, were further explored. While biofilm inhibition and disruption were demonstrated *in vitro*, the relevance of these findings to *in vivo* conditions (e.g., respiratory tract biofilms) is unclear without corroborating *in vivo* biofilm studies. Exploring additional ex vivo models and including female mice in future experiments would further strengthen the study’s applicability. In summary, while the current study provides valuable insights into the potential of phage pK2420 as a therapeutic agent, further research is needed to address these limitations and fully evaluate the clinical potential of this phage therapy.

### Conclusions

Our findings demonstrated that bacteriophage PK2424 exhibits promising biosafety and therapeutic efficacy against hvKp-induced pulmonary infections and dissemination in a murine model. These results suggest that phage PK2424 may be a potential option for the treatment of hvKp infections. Furthermore, this study examined interactions among *K. pneumoniae*, bacteriophages, and the host, providing a valuable reference for research into phage pharmacokinetics. However, further studies are required to address the limitations identified, including evaluation of broader serotype coverage, optimization of dosing regimens, and exploration of resistance mechanisms. Additionally, comprehensive *in vivo* studies are needed to elucidate the long-term effects of phage therapy, interactions with the host immune system, and its impact on the microbiome. Addressing these gaps will be crucial to advancing the clinical translation of phage PK2420 and similar therapeutic agents.

## Data Availability

The whole-genome sequence of phage PK2420 in this study has been deposited in the NCBI (GenBank accession number PQ589833). Data are available in supplemental material: the survival rates of K2420-infected mice, string test result, biofilm formation, genome, virulence factors, and antibiotic resistance gene analysis of K2420; lytic efficiency, host range determination, sensitivity analysis for *K. pneumoniae* isolates, and genome analysis of PK2420; and rates of *K. pneumoniae* detection in mouse lung tissue.

## SUPPLEMENTAL MATERIAL

Supplemental figures msystems.01632-24-s0001.pdf Fig. S1 to S3.

Supplemental tables msystems.01632-24-s0002.xlsx Tables S1 to S6.

ASM does not own the copyrights to Supplemental Material that may be linked to, or accessed through, an article. The authors have granted ASM a non-exclusive, world-wide license to publish the Supplemental Material files. Please contact the corresponding author directly for reuse.
