# Supplementary material for: Genomic analysis and therapeutic efficacy evaluation of bacteriophage PK2420 for pneumonia caused by hypervirulent Klebsiella pneumoniae (K20 serotype)
Source: mSystems. 2025 Apr 16;10(5):e01632-24. doi: 10.1128/msystems.01632-24 (PMC12090786; doi:10.1128/msystems.01632-24)
Supplement: Supplemental figures — Fig. S1 to S3. [file msystems.01632-24-s0001.pdf]

Figure S1

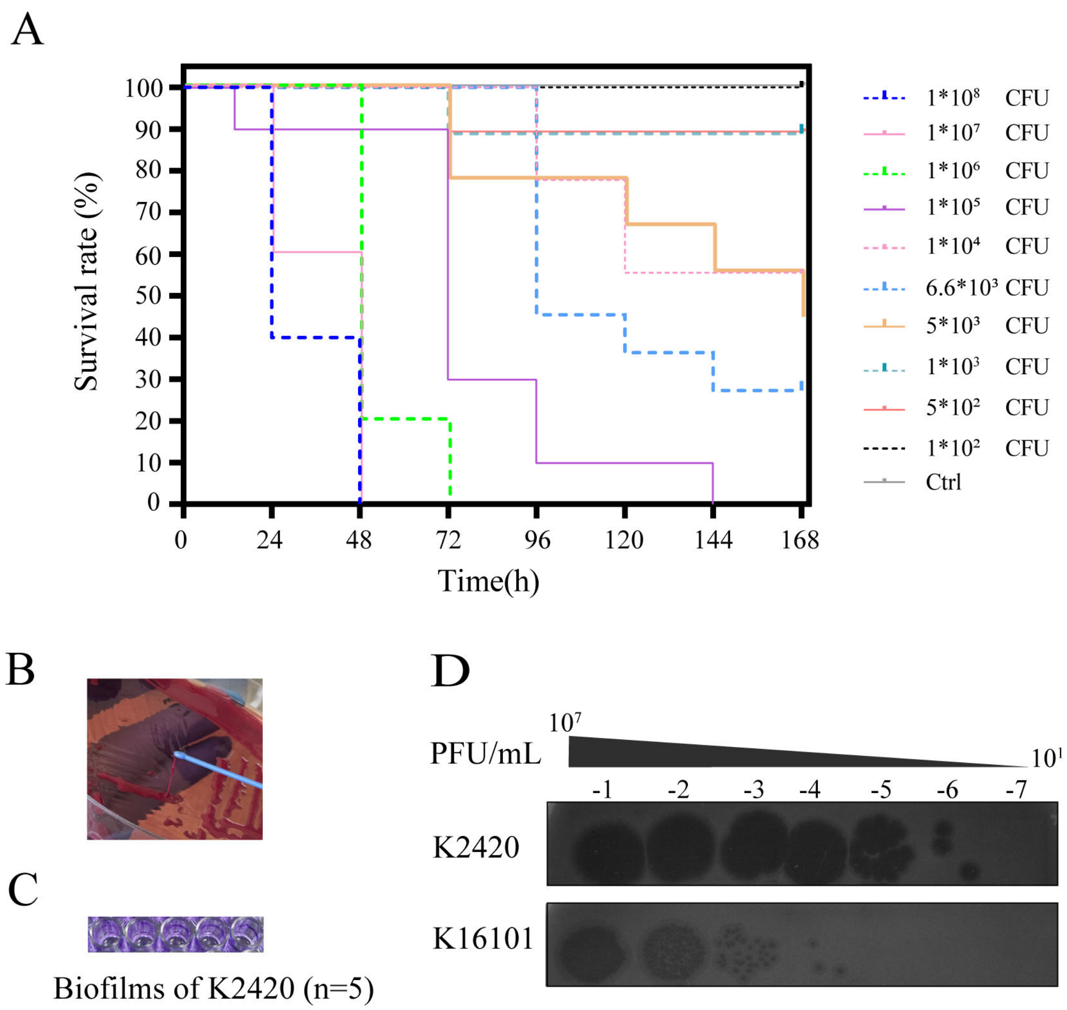

(A) Survival rates of *K. pneumoniae*-infected mice after intratracheal injections. (B) String test results for K2420. (C) Biofilm formation by K2420, visualized using crystal violet staining. (D) Lytic efficiency of PK2420 against *K. pneumoniae* strains K2420 and K16101.

Figure S2

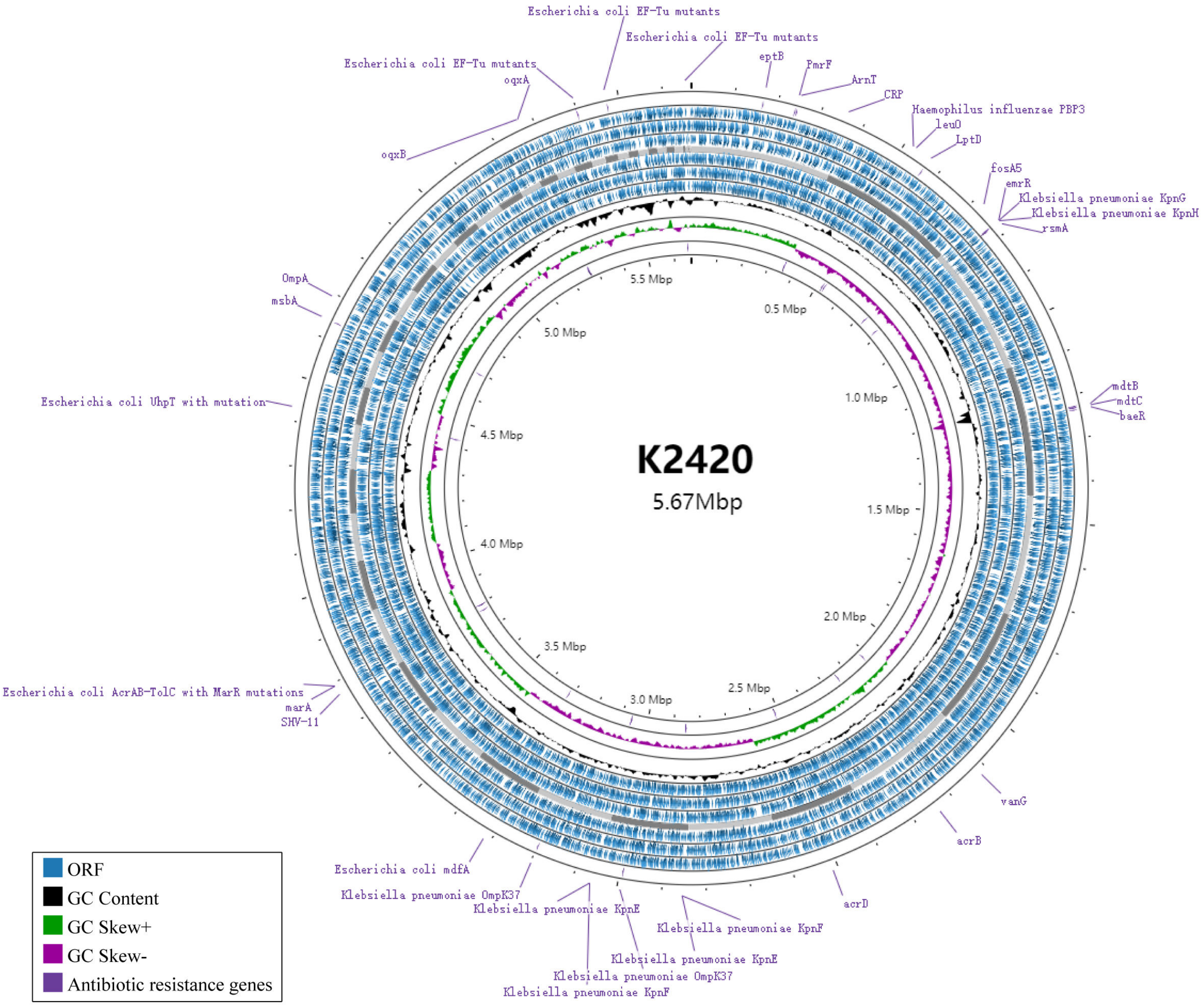

Analysis of K2420 genome and antibiotic resistance genes.

Figure S3

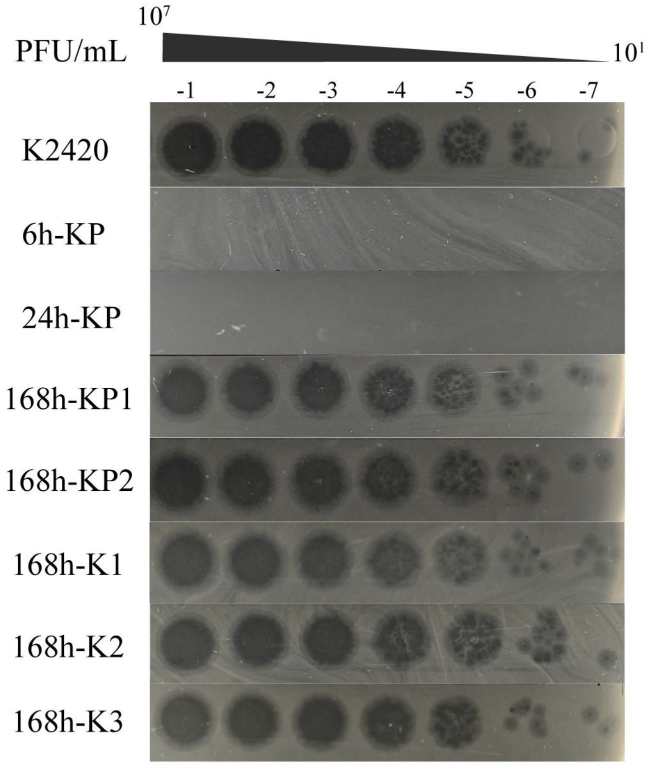

Phage PK2420 sensitivity analysis for *K. pneumoniae* isolates.
